# Stimulation of PBMC and Monocyte-Derived Macrophages *via* Toll-Like Receptor Activates Innate Immune Pathways in HIV-Infected Patients on Virally Suppressive Combination Antiretroviral Therapy

**DOI:** 10.3389/fimmu.2016.00614

**Published:** 2016-12-19

**Authors:** Esther Merlini, Camilla Tincati, Mara Biasin, Irma Saulle, Federico Angelo Cazzaniga, Antonella d’Arminio Monforte, Amedeo J. Cappione, Jennifer Snyder-Cappione, Mario Clerici, Giulia Carla Marchetti

**Affiliations:** ^1^Department of Health Sciences, Clinic of Infectious Diseases, ASST Santi Paolo e Carlo, University of Milan, Milan, Italy; ^2^Department of Biomedical and Clinical Sciences – “L. Sacco”, University of Milan, Milan, Italy; ^3^EMD Millipore, Danvers, MA, USA; ^4^Flow Cytometry Core, Boston University, Boston, MA, USA; ^5^Department of Physiopathology and Transplants, University of Milan, Milan, Italy; ^6^Don C. Gnocchi Foundation, Istituti di Ricovero e Cura a Carattere Scientifico (IRCCS), Milan, Italy

**Keywords:** TLR pathway, immunological non-responders, HIV-1, cART, immune activation

## Abstract

In HIV-infected, combination antiretroviral therapy (cART)-treated patients, immune activation and microbial translocation persist and associate with inadequate CD4 recovery and morbidity/mortality. We analyzed whether alterations in the toll-like receptor (TLR) pathway could be responsible for the immune hyperactivation seen in these patients. PBMC/monocyte-derived macrophages (MDMs) of 28 HIV+ untreated and 35 cART-treated patients with HIV-RNA < 40 cp/mL [20 Full Responders (FRs): CD4 ≥ 350; 15 Immunological Non-Responders (INRs): CD4 < 350], as well as of 16 healthy controls were stimulated with a panel of TLR agonists. We measured: CD4/CD8/CD14/CD38/HLA-DR/Ki67/AnnexinV/CD69/TLR4/8 (Flow Cytometry); PBMC expression of 84 TLR pathway genes (qPCR); PBMC/MDM cytokine release (Multiplex); and plasma lipopolysaccharide (LPS)/sCD14 (LAL/ELISA). PBMC/MDM from cART patients responded weakly to LPS stimulation but released high amounts of pro-inflammatory cytokines. MDM from these patients were characterized by a reduced expression of HLA-DR+ MDM and failed to expand activated HLA-DR+ CD38+ T-lymphocytes. PBMC/MDM from cART patients responded more robustly to ssRNA stimulation; this resulted in a significant expansion of activated CD38 + CD8 and the release of amounts of pro-inflammatory cytokines comparable to those seen in untreated viremic patients. Despite greater constitutive TLR pathway gene expression, PBMC from INRs seemed to upregulate only type I IFN genes following TLR stimulation, whereas PBMC from full responders showed a broader response. Systemic exposure to microbial antigens drives immune activation during cART by triggering TLRs. Bacterial stimulation modifies MDM function/pro-inflammatory profile in cART patients without affecting T-lymphocytes; this suggests translocating bacteria as selective stimulus to chronic innate activation during cART. High constitutive TLR activation is seen in patients lacking CD4 recovery, suggesting an exhausted immune *milieu*, anergic to further antigen encounters.

## Introduction

Virally effective combination antiretroviral therapy (cART) is characterized by persistent immune hyperactivation, which has been proven a potent determinant of impaired immune recovery (1–4) and non-AIDS morbidity/mortality (5–9), urging the identification of causative pathways. Indeed, a convincing body of data has accumulated that indicates immune activation not only as a consequence of viral-specific challenge but also as a reflection of bystander activation, resulting from innate immune responses (10, 11).

Microbial and viral components are known to trigger the innate immune response *via* toll-like receptor (TLR) signaling (12). In turn, TLR-driven cytokine production from monocytes/macrophages and dendritic cells prompts T-cell activation, thus establishing the adaptive immune response (13–18). In untreated HIV infection, altered TLR expression and responsiveness have been described (19–21) and are only partially normalized by cART (21). Indeed, HIV-1 encodes for various TLR7/8 ligands that can mediate direct activation of the immune system *in vitro* (22–24). Likewise, HIV-driven gut barrier damage is not reverted by cART (25–28) and leads to the passage of microbial products in peripheral blood, mainly lipopolysaccharide (LPS), which is a TLR4 agonist (29, 30). Circulating LPS levels have been associated with immune activation both in treated and untreated HIV (31–35); furthermore, exogenous *in vivo* LPS administration has been described to enhance immune activation (34). Besides, recent literature in both HIV-negative and HIV-positive individuals provided *ex vivo* evidence for a direct role of translocating microbial products in driving immune activation. In particular, *ex vivo* stimulation of PBMCs and antigen-presenting cells with bacterial ligands (including LPS), commensal bacteria, and combined bacterial and viral stimulus results in the production of pro- and anti-inflammatory cytokines (36–50). In cART-treated patients, increased CD8+ CD38+ cells have been reported upon LPS exposure in subjects with poor CD4+ T-cell restoration (50) as well as impaired IFN-α production, following stimulation of plasmacytoid dendritic cells with TLR7 and TLR9 agonists (51).

These data would altogether imply the testable hypothesis of TLR pathway as mediator of persistent immune activation/inflammation upon effective cART. However, a thorough investigation of the contribution of TLR pathway in sustaining immune activation in HIV+ patients on virologically suppressive cART as compared to both HIV+ untreated and uninfected individuals, and whether it might be associated to poor CD4 recovery on cART, has not been established yet.

To bridge this gap, we determined the effect of TLR challenge on downstream pathways in T-lymphocytes and monocytes/macrophages from HIV-infected cART-untreated and treated individuals with different degrees of immune reconstitution who had evidence of microbial translocation and compared them to uninfected controls.

## Patients and Methods

### Patients

Sixty-three HIV-infected individuals were consecutively enrolled at the Clinic of Infectious Diseases and Tropical Medicine, ASST Santi Paolo e Carlo, University of Milan, Italy. Thirty-five patients were on stable cART for at least 12 months, with undetectable plasma HIV-RNA load (<40 cp/mL) in at least two consecutive assessments and CD4 nadir ≤350/mmc. Twenty-eight patients were antiretroviral naïve, with any CD4 count. Individuals with either signs/symptoms of gastrointestinal diseases or on antibiotic therapy at the time of study were excluded. HIV+ on cART were divided into two groups according to the degree of immune reconstitution following the introduction of cART: Full Responders (FRs, *n* = 20) with CD4+ ≥350/mmc and Immunological Non-Responders (INRs, *n* = 15) with CD4+ <350/mmc. We also enrolled 16 HIV-negative healthy subjects as controls.

All of the enrolled patients provided written informed consent according to the Ethical Committee of our Institution (Comitato Etico, ASST “Santi Paolo e Carlo”, Milan, Italy). The ethics committee specifically approved this study. All subjects gave written informed consent in accordance with the Declaration of Helsinki.

### Isolation and Culturing of Primary Monocytes to Obtain Monocyte-Derived Macrophages

Monocytes from Ficoll-isolated PBMCs were separated from lymphocytes by adherence to tissue culture-treated plates (52). After 48 h of incubation, non-adherent cells were removed *via* two washes with warm RPMI. The purity of the monocytes was >90%, as determined by immunofluorescent staining with antiCD14 FITC antibody (BD Pharmigen, San Diego, CA, USA). The monocytes were differentiated into macrophages (MDMs) by culturing in RPMI medium supplemented with 10% fetal bovine serum, 2 mM glutamine, 100 U of penicillin/ml, and 100 µg of streptomycin/ml for 15 days prior to stimulation. The monocyte-derived macrophages (MDMs) were washed with phosphate-buffered saline (PBS), and the culture medium was replaced every 2 days. After 15 days, the MDMs were removed by gently scraping with a plastic cell scraper and cold PBS.

### Stimulation of PBMCs and MDMs

Dose–response and timing curves were performed for each stimulus. Ficoll-separated PBMCs (4 × 10^6^ cells/well) and MDMs (5 × 10^5^ cells/well) were stimulated for 24 h with LPS (50 µg/mL), peptidoglycan (PGN) (10 µg/mL), lipoteichoic acid (LTA) (1 µg/mL), ssRNA40, a uridine-rich ssRNA analog of HIV-1 ssRNA (6,25 µg/mL) (InvivoGen, San Diego, CA, USA), interferon-γ (IFNγ) (100 U/mL), anti-CD28 (1.25 µg/mL) (R&D System, Minneapolis, MN, USA), and anti-CD3 (2.5 µg/mL) (BD Pharmigen, San Diego, CA, USA). After 24 h, the cells were harvested for flow cytometry analyses; the supernatants of a subset of 5 HIV−, 5 HIV+ untreated, and 11 HIV + cART (5 INRs and 6 FRs) patients were collected and stored for the Luminex assay.

### Flow Cytometry

Cell-surface molecule expression of the cultured PBMCs and MDMs was evaluated by flow cytometry (FC500, Beckman Coulter) using the following fluorochrome-labeled antibodies: CD4-PECy7, CD8-PECy5, CD14-PECy7, CD69-PECy5, CD45-PE, CD3-PECy7 (Beckman Coulter, Milan, Italy), CCR7-PE, CD45RA-FITC, HLA-DR-FITC, CD38-PE, CD4-PE, CD8-PerCPCy5.5, Ki67-FITC, CD14-FITC (BD Bioscience, San Diego, CA, USA), TLR-4-PE (R&D Systems, Minneapolis, MN, USA), and TLR-8-PE (Thermoscientific, Tema Ricerca, Milan, Italy). Activation (HLA-DR and CD38), apoptosis (Annexin V), proliferation (Ki67), maturation (CCR7/CD45RA), and TLR expression (TLR-4,-8) were determined from the total PBMC cultures. MDM activation and function markers were measured by the expression of CD69 and HLA-DR, respectively.

The following reagent combinations were used: CD8/CD4/CD38/HLA-DR, CD4/AnnexinV/7AAD, CD8/AnnexinV/7AAD, CD8/CD4/Ki67, CD45RA/CCR7/CD4/CD8, CD45/CD14/CD69/HLA-DR, CD3/CD14/TLR4, and CD3/CD14/TLR8. CXP software from Beckman Coulter was used for the analyses. PBMCs were gated first based on side- and forward-scatter properties, then as CD4+ or CD8+ and finally as CD38/HLA-DR or Annexin V/7AAD or Ki67 or CCR7/CD45RA. To measure TLR expression, PBMCs were first gated based on side- and forward-scatter properties, then as CD3/CD14 and, within the CD14+ gate, cells were gated as TLR4+ or TLR8+. MDMs were gated first based on side-scatter properties and CD45+, then as CD14+, and finally as CD69/HLA-DR.

### Microbial Translocation Markers

Plasma levels of sCD14 and LPS were measured by an ELISA assay (R&D Systems, Minneapolis, MN, USA) and the LAL test (Kinetic-QCL; Bio Whittaker, Walkersville, MD, USA), respectively, and used according to the manufacturer’s instructions.

### HIV-RNA Quantification

Plasma HIV-RNA was quantified using the Abbott Real*Time* HIV-1 assay, with a detection limit of 40 cp/ml, according to manufacturer’s instruction. The HIV-RNA copy numbers between 0 and 40 (low-level residual viremia) were extrapolated from the standard curve of the assay (Abbott Laboratories, Princeton, NJ, USA).

### Multiplex Assays

The relative contents of 25 analytes in culture supernatants of a subset of 11 HIV+ cART-treated subjects (5 INRs and 6 FRs) were determined using the human Th17 Magnetic Bead Panel (EMD Millipore; Billerica, MA, USA) with a Bio-Plex^®^ MAGPIX™ Multiplex Reader (Bio-Rad Laboratories, Hercules, CA, USA), according to the manufacturer’s instructions. The relative content of 12 analytes in culture supernatants of a subset of five HIV + naïve subjects and five HIV-negative healthy controls was quantified using Luminex Screening Assay (R&D Systems, Minneapolis, MN, USA) with a Bio-Plex^®^ MAGPIX™ Multiplex Reader (Bio-Rad Laboratories, Hercules, CA, USA). All samples were run in duplicate. The raw data were analyzed using Bio-Plex Manager software. Standard curves were generated from lyophilized standards provided with each kit. The concentration for each analyte in each sample was determined *via* interpolation from each corresponding standard curve.

### RNA Extraction and Reverse Transcription

RNA was extracted from cultured PBMCs using the acid guanidium thiocyanate–phenol–chloroform method. RNA was purified from genomic DNA with RNase-free DNase (RQ1 DNase, Promega, Madison, WI, USA). Then, 1 µg of RNA was reverse transcribed into first-strand cDNA in a 20-µL final volume containing 1 µM random hexanucleotide primers, 1 µM oligo dT, and 200 U Moloney murine leukemia virus reverse transcriptase (Clontech, Palo Alto, CA, USA).

### TLR Signaling Pathway

The TLR signaling pathway was analyzed in a PCR array that include a set of 84 optimized real-time PCR primers plus five housekeeping genes on a 96-well plates; the procedures suggested by the manufacturer were followed (SABiosciences Corporation, Frederick, MD, USA). Controls for genomic DNA contamination, RNA quality, and general PCR performance were also included on each array. The experiments were run on all HIV-negative healthy controls, HIV + untreated, and cART (INRs and FRs) subjects included in the study and pooled into unique HIV-negative, untreated HIV+, HIV + cART (INRs and FRs) samples, respectively (53). Thus, the results represent the mean value of the different targets analyzed in the study groups. Furthermore, the targets with marked differences between groups were retested by real-time PCR on each individual sample to confirm the data obtained in the array (data not shown).

### Statistical Analysis

All continuous variables are presented as medians and interquartile ranges (25th–75th percentile), whereas categorical data are shown as absolute numbers and percentages. Chi- squared or Fisher’s Exact test was used for the analyses of categorical variables. Mann–Whitney *U* test, Wilcoxon test, and Kruskal–Wallis test were used for the comparison of immunological parameters between the study groups. Correction for multiple comparisons was performed by *post hoc* analysis (Bonferroni or Dunn test). *p*-Values < 0.05 were considered significant. Statistical analyses and graphs were performed using GraphPad Prism 5 software.

## Results

### Study Population Characteristics

The epidemiological, clinical, and immunological characteristics of the HIV-infected study groups and HIV-uninfected healthy controls are listed in Table 1. While all cART patients had an HIV-RNA < 40 cp/ml (as per inclusion criteria), median HIV low-level residual viremia was 20 cp/ml (IQR: 15–22). As shown in Table 1, cART patients featured lower CD4 nadir (*p* < 0.0001) and CD4 count at time of analysis (*p* = 0.003), displayed higher proportion of AIDS diagnosis (*p* = 0.003) and longer HIV infection (*p* < 0.0001).

**Table 1:** **Epidemiological, clinical, and immunological characteristics of the study groups**.

	HIV-negative (*n* = 16)	HIV+ untreated (*n* = 28)	HIV+ cART (*n* = 35)	*p*-Value
Age, years (IQR)^a^	31 (28–35)	34 (29–39)	48 (41–64)	**<0.0001**
Sex (%)^b^				**<0.0001**
Female	11 (69)	3 (11)	7 (20)	
Risk factors (%)^b^				**<0.0001**
Heterosex	13 (81)	6 (21)	18 (51)	
Homosex/bisex	3 (19)	22 (79)	11 (31)	
IDU	0 (0)	0 (0)	6 (18)	
HCV co-infection (%)^b^				0.231
Yes	0(0)	2 (7)	5 (14)	
AIDS diagnosis^b^ (%) (yes)	N/A	2 (7)	14 (40)	**0.003**
Time since first HIV Ab+ test, years (IQR)^a^	N/A	2 (2–4)	5 (4–7)	**<0.0001**
CD4 T-cell count (IQR)^a^				
Nadir (*n*)	N/A	400 (324–524)	94 (26–217)	**<0.0001**
Time of analysis (*n*)		521 (413–592)	372 (253–455)	**0.003**
HIV-RNA Log cp/mL (IQR)^a^ at time of analysis	N/A	4.39 (3.74–5.12)	1.59 (1.59–1.59)	**<0.0001**
Low-level residual viremia HIV-RNA (cp/ml)	N/A	N/A	20 (15–22)	N/A
cART duration, years (IQR)^a^	N/A	N/A	5 (3–6)	N/A
cART regimen (%)^b^				
NRTI + PI		N/A	24 (68.5)	N/A
NRTI + NNRTI	N/A		8 (23)	
Others			3 (8.5)	
TLR4+ CD14+ (%) (IQR)	98.6 (97–99)	89.3 (47–99)	78 (57–87)	**0.002**
TLR8+ CD14+ (%) (IQR)	99.8 (99.7–100)	99.4 (97–100)	90.2 (84–96)	**0.001**
LPS, pg/ml (IQR)	75 (75–81)	187 (97–427)	203 (83–258)	**0.0045**
sCD14, μg/ml (IQR)	1.96 (1.39–2.10)	3.32 (2.81–5.14)	5.61 (3.25–7.92)	**0.0002**
CD45RA+ CCR7+ CD4+ (%) (IQR)	49.9 (2.4–57.4)	2.1 (1.1–22.7)	2.6 (1.9–8.8)	0.102
CD45RA− CCR7+ CD4+ (%) (IQR)	18.1 (3.6–23.2)	3.4 (2.1–8.1)	2.77 (1.7–11.7)	**0.035**
CD45RA− CCR7− CD4+ (%) (IQR)	21.1 (12.2–54.7)	48.6 (33.8–58.7)	45.4 (27.1–66.3)	0.242
CD45RA+ CCR7− CD4+ (%) (IQR)	5.8 (3.4–31.3)	36.6 (22.4–55.9)	32.3 (22.1–49.6)	**0.026**
CD45RA+ CCR7+ CD8+ (%) (IQR)	23.4 (12.9–24.6)	12.3 (4.5–46.1)	6.5 (2.6–17.9)	0.254
CD45RA− CCR7+ CD8+ (%) (IQR)	1.3 (1.1–2.2)	1.3 (0.6–7.3)	2.1 (0.8–5.7)	0.376
CD45RA− CCR7− CD8+ (%) (IQR)	25.1 (18.9–36.7)	49.1 (19.1–51.7)	36.2 (18.7–58.3)	0.494
CD45RA+ CCR7− CD8+ (%) (IQR)	43.1 (39.1–59.1)	35.4 (27.6–44.3)	42.9 (27.5–56.9)	0.727

*^a^Data are median (IQR). IQR, interquartile range; statistical analyses: Mann–Whitney *U* Test or Kruskall–Wallis with Dunn’s Multiple Comparison test*.

*^b^Data are *n* (%), statistical analyses: Pearson chi squared or Fisher exact test; *p* values are referred to the comparison between the three study groups where applicable*.

*cART, combination of antiretroviral therapy; IDU, intravenous drug users; NRTI, nucleoside reverse transcriptase inhibitor; PI, protease inhibitor; NNRTI, non-nucleoside reverse transcriptase inhibitor; LPS, lipopolysaccharide; TLR, toll-like receptors*.

*Statistically significant *p* values (*p* < 0.05) are highlighted in bold font*.

Increased microbial translocation with HIV infection is evident as both cART and untreated HIV+ subject groups have, on average, higher LPS/sCD14 levels in the plasma than HIV-uninfected controls (LPS: *p* = 0.0045; sCD14: *p* = 0.0002). Furthermore, both HIV+ untreated and cART patients have skewed CD4 T-cell immune phenotypes, with lower frequencies of central memory (*p* = 0.035) and higher frequencies of terminally differentiated (TD) CD4+ T cells (*p* = 0.026) as compared to healthy controls.

Table 2 summarizes the characteristics of HIV+ cART individuals, which were stratified into two groups FRs (*n* = 20) and INRs (*n* = 15) according to the degree of immune recovery. Patients were comparable for all epidemiological and immunological features. No differences in T-cell maturation phenotypes and microbial translocation markers were found between the groups (Table 2).

**Table 2:** **Epidemiological, clinical, and immunological characteristics of the study groups**.

	FRs (*n* = 20)	INRs (*n* = 15)	*p*-Value
Age, years (IQR)^a^	51 (41–68)	45 (39–54)	0.278
Sex (%)^b^			
Female	6 (30)	1 (7)	0.198
Risk factors (%)^b^			0.271
Heterosex	11 (55)	7 (47)	
Homosex/bisex	6 (30)	5 (33)	
IDU	3 (15)	3 (20)	
HCV co-infection (%)^b^			0.722
Yes	3 (15)	2 (13)	
AIDS diagnosis^b^ (%) (yes)	5 (25)	9 (60)	0.079
Time since first HIV Ab+, years (IQR)^a^	5 (5–7)	5 (3–7)	0.552
CD4 T-cell count (IQR)^a^			
Nadir (*n*)	97 (42–230)	94 (26–124)	0.342
Time of analysis (*n*)	451 (404–585)	237 (164–299)	**<0.0001**
HIV-RNA Log cp/mL (IQR)^a^ at time of analysis	1.59 (1.59–1.59)	1.59 (1.59–1.59)	0.901
Low-level residual viremia HIV-RNA, cp/ml	20 (14–21)	20 (17–28)	0.321
cART duration, years (IQR)^a^	4.5 (3–5)	5 (3–6)	0.444
cART regimen (%)^b^			0.112
NRTI + PI	11 (55)	13 (86)	
NRTI + NNRTI	7 (35)	1 (7)	
Others	2 (10)	1 (7)	
Plasma LPS, pg/mL (IQR)^a^	146 (75–264)	235 (108–258)	0.447
Plasma sCD14, μg/mL (IQR)^a^	6.36 (3.5–7.98)	4.23 (2.84–5.99)	0.139
Toll-like receptor (TLR) 4+ CD14+, % (IQR)	79.5 (62.8–86.1)	77.7 (44.3–90.5)	0.680
TLR8+ CD14+, % (IQR)	84.9 (69.7–93.8)	80.2 (76.1–87.8)	0.770
CD45RA+ CCR7+ CD4+ (%) (IQR)	3.4 (2.4–53.3)	2.4 (0.7–7.7)	0.231
CD45RA− CCR7+ CD4+ (%) (IQR)	3.7 (1.5–18.8)	2.5 (1.8–4.4)	0.536
CD45RA− CCR7− CD4+ (%) (IQR)	42.1 (13.8–45.7)	58.8 (42.3–67.5)	0.107
CD45RA+ CCR7− CD4+ (%) (IQR)	23.1 (4.3–53.2)	35.5 (26.8–47.4)	0.649
CD45RA+ CCR7+ CD8+ (%) (IQR)	6.3 (2–37.3)	5.2 (2.9–10.6)	0.837
CD45RA− CCR7+ CD8+ (%) (IQR)	3.8 (0.5–6.9)	1.7 (0.5–6.8)	0.620
CD45RA− CCR7− CD8+ (%) (IQR)	36.2 (9.7–54.1)	42.5 (16.5–58.4)	0.649
CD45RA+ CCR7− CD8+ (%) (IQR)	42.6 (26–56.2)	40.4 (24.7–55.3)	0.901

*^a^Data are median (IQR). IQR, interquartile range; statistical analyses: Mann–Whitney *U* Test*.

*^b^Data are *n* (%), statistical analyses: Pearson chi squared or Fisher exact test; FRs: full responders (CD4 ≥ 350/mmc; HIV-RNA < 40 cp/mL); INRs: immunological non-responders (CD4 < 350/mmc; HIV-RNA < 40 cp/mL)*.

*IDU, intravenous drug users; LPS, lipopolysaccharide; cART, combination of antiretroviral therapy; NRTI, nucleoside reverse transcriptase inhibitor; PI, protease inhibitor; NNRTI, non-nucleoside reverse transcriptase inhibitor*.

*Statistically significant *p* values (*p* < 0.05) are highlighted in bold font*.

### Effect of TLR Stimulation on PBMC

We first investigated possible differences in the response of PBMC to TLR stimulation in HIV-infected untreated and treated patients versus uninfected controls.

#### *In Vitro* TLR Challenge on PBMCs: T Cell Activation, Proliferation, and Apoptosis

**Figure 1:**
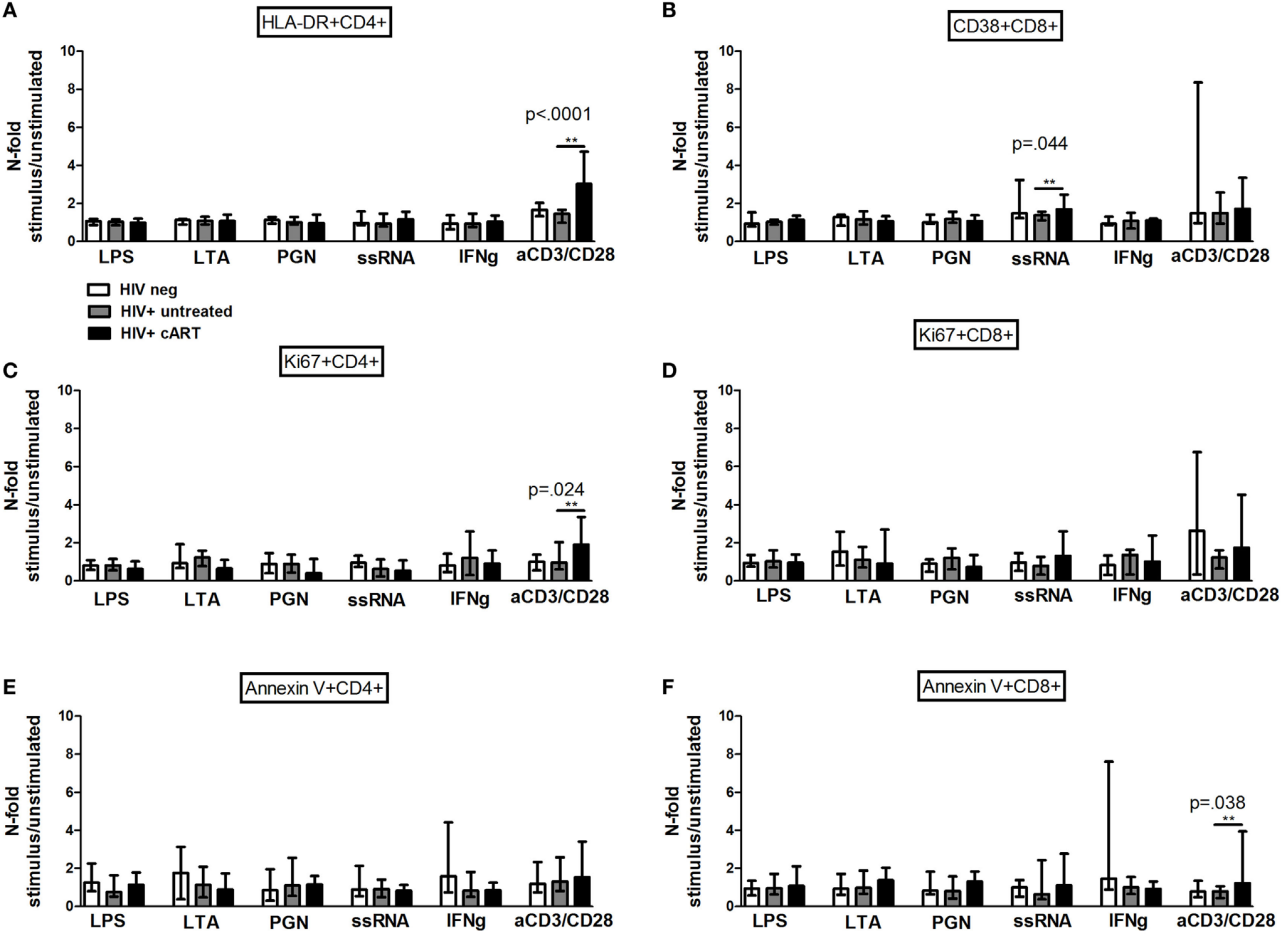
***In vitro* toll-like receptor (TLR) challenge on PBMCs: CD4 and CD8 T-cell activation, proliferation, and apoptosis**. Data shown as a fold change: (stimulated percentage value)/(unstimulated percentage value). *indicates which comparison is the most significant **(A)** No upregulation of HLA-DR on CD4 T-cells in all the three study groups following TLR challenge [lipopolysaccharide (LPS) *p* = 0.754; lipoteichoic acid (LTA) *p* = 0.945; peptidoglycan (PGN) *p* = 0.739; ssRNA *p* = 0.623; IFNγ *p* = 0.396; aCD3/CD28 *p* < 0.0001]. **(B)** Significant increase of CD38 + CD8 T-cell upon viral challenge alone in combination antiretroviral therapy group (LPS *p* = 0.081; LTA *p* = 0.587; PGN *p* = 0.360; ssRNA *p* = 0.044; IFNγ *p* = 0.764; aCD3/CD28 *p* = 0.304). **(C,D)** No differences in the proportion of Ki67 + CD4 T-cells (LPS *p* = 0.193; LTA *p* = 0.083; PGN *p* = 0.149; ssRNA *p* = 0.358; IFNγ *p* = 0.762; aCD3/CD28 *p* = 0.024) and Ki67 + CD8 T-cells following the exposure to TLR ligands (LPS *p* = 0.609; LTA *p* = 0.549; PGN *p* = 0.317; ssRNA *p* = 0.237; IFNγ *p* = 0.507; aCD3/CD28 *p* = 0.069). **(E,F)** No changes in pro-apoptotic Annexin V + CD4 (LPS *p* = 0.268; LTA *p* = 0.638; PGN *p* = 0.672; ssRNA *p* = 0.685; IFNγ *p* = 0.491; aCD3/CD28 *p* = 0.672) and CD8 (LPS *p* = 0.606; LTA *p* = 0.503; PGN *p* = 0.149; ssRNA *p* = 0.335; IFNγ *p* = 0.251; aCD3/CD28 *p* = 0.038) T-cells.

The effect of TLR stimulation on T cell activation, proliferation, and apoptosis was first determined. Interestingly, while the frequencies of activated HLA-DR+ CD4 and CD38 + CD8, proliferating Ki67+ and pro-apoptotic Annexin V + CD4, and CD8 T-cell subpopulations were not affected by bacterial stimulation in all study groups (Figures 1A–F), activated CD38 + CD8 T-cells were expanded by viral stimulation uniquely in cART-treated patients who reached significantly higher proportions than the other groups (ssRNA *p* = 0.044; Figure 1B).

**Figure 2:**
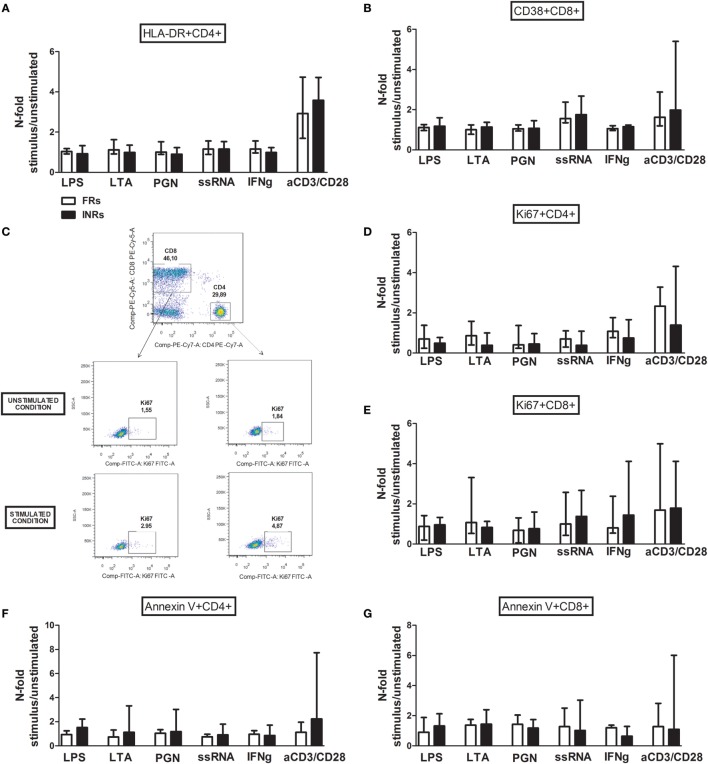
***In vitro* toll-like receptor (TLR) challenge on PBMCs: CD4 and CD8 T-cell activation, proliferation, and apoptosis in Full Responders (FRs) and Immunological Non-Responders (INRs)**. Data shown as a fold change: (stimulated percentage value)/(unstimulated percentage value). **(A)** No upregulation of HLA-DR on CD4 T-cell following TLR stimulation between FRs and INRs [lipopolysaccharide (LPS) *p* = 0.300; lipoteichoic acid (LTA) *p* = 0.271; peptidoglycan (PGN) *p* = 0.258; ssRNA *p* = 0.683; IFNγ *p* = 0.287; aCD3/CD28 *p* = 0.975]. **(B)** No differences in CD38 + CD8 T-cell, according to the degree of immune recovery (LPS *p* = 0.141; LTA *p* = 0.091; PGN *p* = 0.573; ssRNA *p* = 0.442; IFNγ *p* = 0.271; aCD3/CD28 *p* = 0.257). **(C)** Gating strategy for Ki67 measurement in both CD4 and CD8 T-cell subset before and after TLR stimulation (flow cytometry). **(D,E)** No changes in the proportion of Ki67 + CD4 T-cells (LPS *p* = 0.271; lipoteichoic acid (LTA) *p* = 0.240; PGN *p* = 0.896; ssRNA *p* = 0.798; IFNγ *p* = 0.267; aCD3/CD28 *p* = 0.183) and Ki67 + CD8 T-cells following the exposure to TLR ligands (LPS *p* = 0.671; LTA *p* = 0.741; PGN *p* = 0.403; ssRNA *p* = 0.471; IFNγ *p* = 0.182; aCD3/CD28 *p* = 0.929). **(F,G)** No changes in pro-apoptotic Annexin V + CD4 (LPS *p* = 0.182; LTA *p* = 0.237; PGN *p* = 0.387; ssRNA *p* = 0.669; IFNγ *p* = 0.963; aCD3/CD28 *p* = 0.079) and CD8 (LPS *p* = 0.661; LTA *p* = 0.905; PGN *p* = 0.546; ssRNA *p* = 0.813; IFNγ *p* = 0.370; aCD3/CD28 *p* = 0.842) T-cells.

The levels of activated HLA-DR+ CD4 and CD38 + CD8 T-cells (Figures 2A,B), proliferating Ki67+ (Figures 2C–E), and apoptotic Annexin V + CD4 and CD8 T-cells (Figures 2F,G) after TLR stimulation were similar between the FRs and INRs among the cART subjects.

#### *mRNA* Expression of Genes Involved in the TLR-Mediated Pathway

To further investigate the effect of TLR stimulation on PBMCs, we next measured gene transcription and cytokine/chemokine response following exposure to the representative TLR4 and TLR7/8 agonists LPS and ssRNA, respectively. We first screened 84 genes involved in the TLR-activation pathway by real-time PCR array in PBMCs from untreated and treated HIV+ patients (FRs and INRs) and HIV-negative controls.

**Figure 3:**
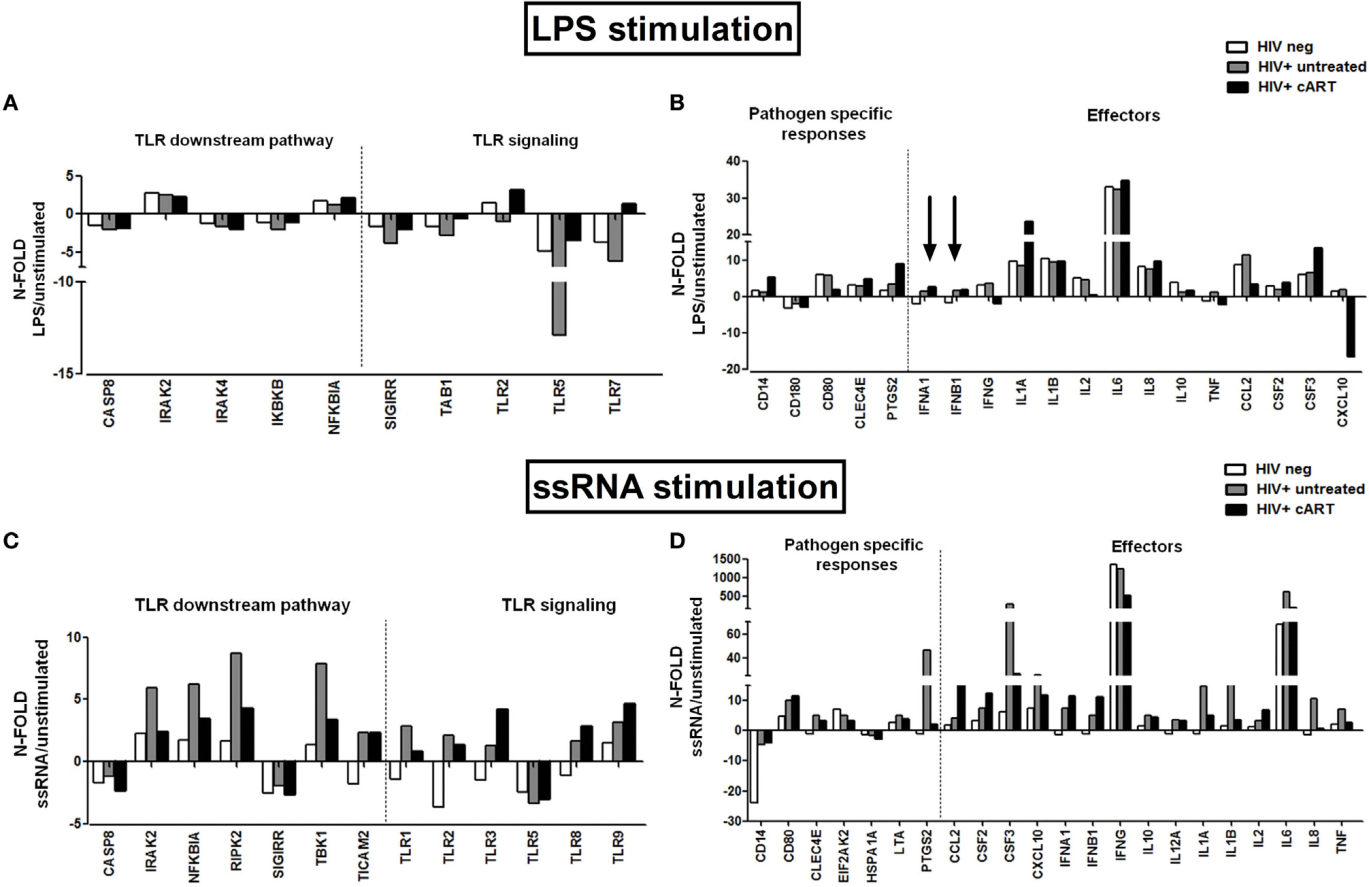
***In vitro* toll-like receptor (TLR) challenge on PBMCs: mRNA expression of genes involved in the TLR pathway**. TLR signaling pathway mRNA expression in HIV-negative healthy controls (white bars), HIV + untreated subjects (gray bars), and HIV + combination antiretroviral therapy (cART) subjects (black bars) for TLR4 and TLR 7/8 stimulation with lipopolysaccharide (LPS) and ssRNA respectively. **(A,B)** Modest upregulation of TLR2, TLR7, CD14, CLE4E, PTGS2, and IL-1α mRNA in HIV + cART patients alone following LPS stimulation. Upregulation of the type I interferon genes (indicated by the arrows) in both treated and untreated HIV-infected patients versus HIV-neg controls. **(C,D)** Higher transcription of IRAK2, NFKBIA, RIPK2, TBK1, CSF3, CXCL-10, IFN-γ, IL-1α, IL-1b, IL-6, IL-8, and TNF-α in untreated HIV+, following viral challenge. Upregulation of CCL2, CSF2, and type I interferons mRNA in cART-treated HIV+ subjects.

In cART-treated patients, LPS stimulation resulted in a modest upregulation of mRNA transcription of TLR2 and TLR7 that was not seen in untreated HIV+ patients (Figure 3A), of genes involved in the pathogen-specific response (CD14, CLEC4E, PTGS2), and pro-inflammatory cytokines IL-1α, IL-6, and CSF3 (Figure 3B). Interestingly, we observed an upregulation of the type I interferon genes (indicated by the arrows) in both HIV+ untreated and cART group compared to HIV-uninfected controls (Figure 3B).

Viral (ssRNA) stimulation induced a more potent upregulation of TLR-activation pathway in HIV+ subjects as compared to uninfected controls (Figures 3C,D). HIV+ untreated subjects showed highest transcription of genes that modulate NFκB activity (IRAK2, NFKBIA, RIPK2, TBK1; Figure 3C), as well as pro-inflammatory cytokines/chemokines (CSF3, CXCL-10, IFN-γ, IL-1a, IL-1b, IL-6, IL-8, TNF-α; Figure 3D). cART group upregulated mRNA specific for CCL2, CSF2, and type I interferons (Figure 3D).

**Figure 4:**
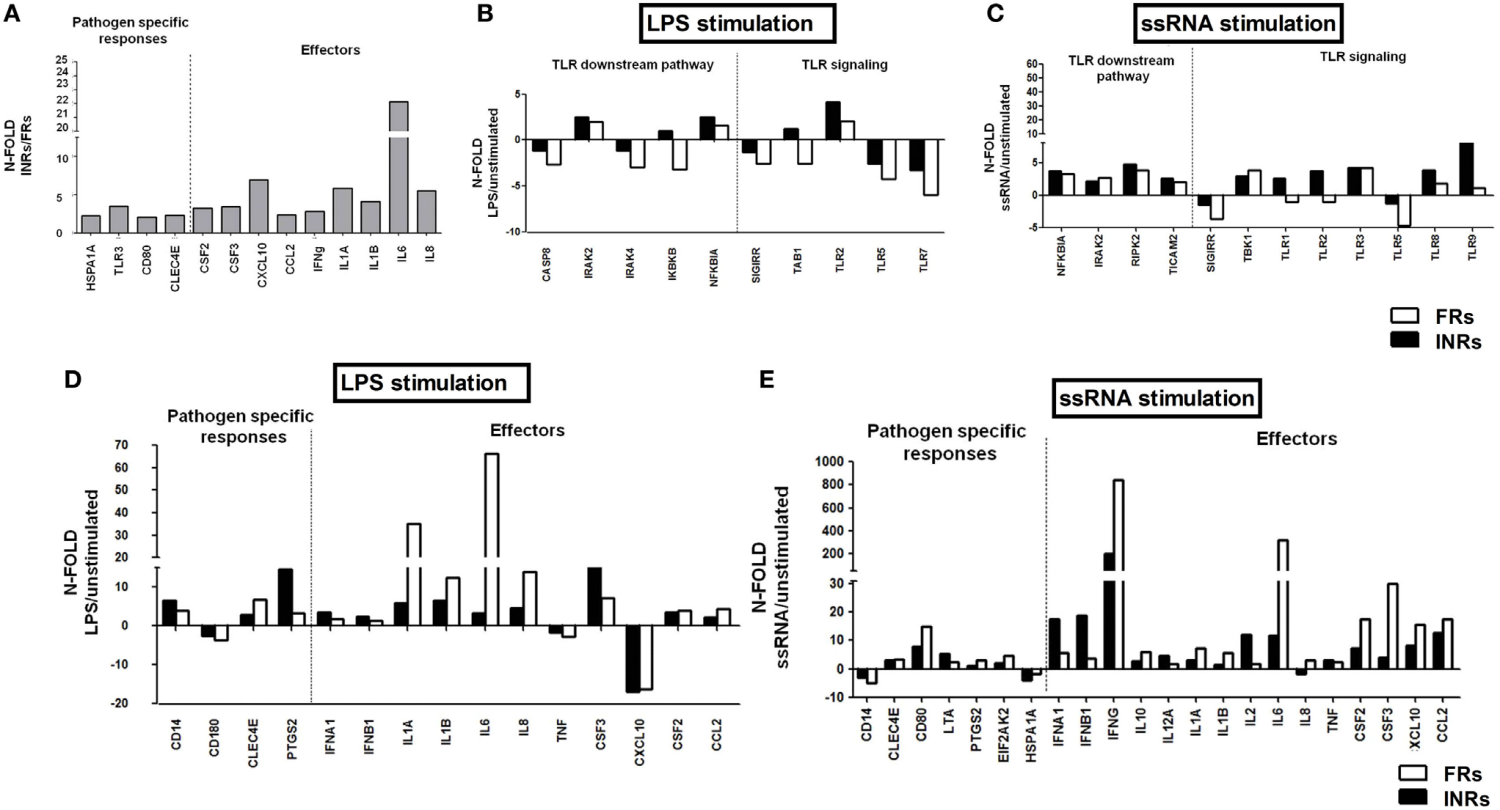
***In vitro* toll-like receptor (TLR) challenge on PBMCs: mRNA expression of genes involved in the TLR pathway in Full Responders (FRs) and Immunological Non-Responders (INRs)**. TLR signaling pathway mRNA expression in HIV + combination antiretroviral therapy (cART) FRs (white bars) and HIV + cART INRs (black bars) for TLR4 and TLR 7/8 stimulation with lipopolysaccharide (LPS) and ssRNA, respectively. **(A)** Higher constitutive mRNA (up to 20-fold) of CCL2, CSF2, CSF3, CXCL10, IFNγ, IL1α, IL1b, IL6, IL8, CLEC4E, HSPA1A, CD80, and TLR3, in INRs as compared to FRs. **(B,C)** No effect on TLR downstream pathway after LPS and ssRNA stimulation. **(D)** Upregulation of IL-1α, IL-6, and IL-8 in FRs alone following LPS stimulation. **(E)** Higher mRNA transcription of IFN-γ, IL-6, CSF2, and CSF3 in FRs and higher type I interferons in INRs after ssRNA stimulation.

Upon a comparative analysis of treated individuals with different response to cART, INRs presented higher constitutive mRNA expression levels (up to 20-fold) of effector molecules, such as CCL2, CSF2, CSF3, CXCL10, IFN-γ, IL1-a, IL-1b, IL-6, IL-8, and of genes involved in the pathogen-specific response (CLEC4E, HSPA1A, CD80, TLR3) (Figure 4A).

Both INRs and FRs failed to show a substantial upregulation of genes involved in the TLR downstream pathway upon LPS and ssRNA exposure (Figures 4B,C).

Interestingly, LPS exposure resulted in the upregulation of IL-1a, IL-6, and IL-8 in FRs alone (Figure 4D). Similarly, FRs were more responsive to ssRNA challenge (IFN-γ, IL-6, CSF2, and CSF3), except for type I interferons, which were higher in INRs (Figure 4E).

These data suggest a hyper-responsiveness to viral stimulation in both untreated and cART-treated HIV-infected subjects, confirming ssRNA as a potent immune activator in the setting of HIV infection. These results also suggest that despite an higher basal expression of genes involved in the TLR pathway, INRs are less responsive to TLR-specific stimulation and show upregulation of only type I IFNs following microbial challenge.

#### Cytokine/Chemokine Release in Supernatants

**Figure 5:**
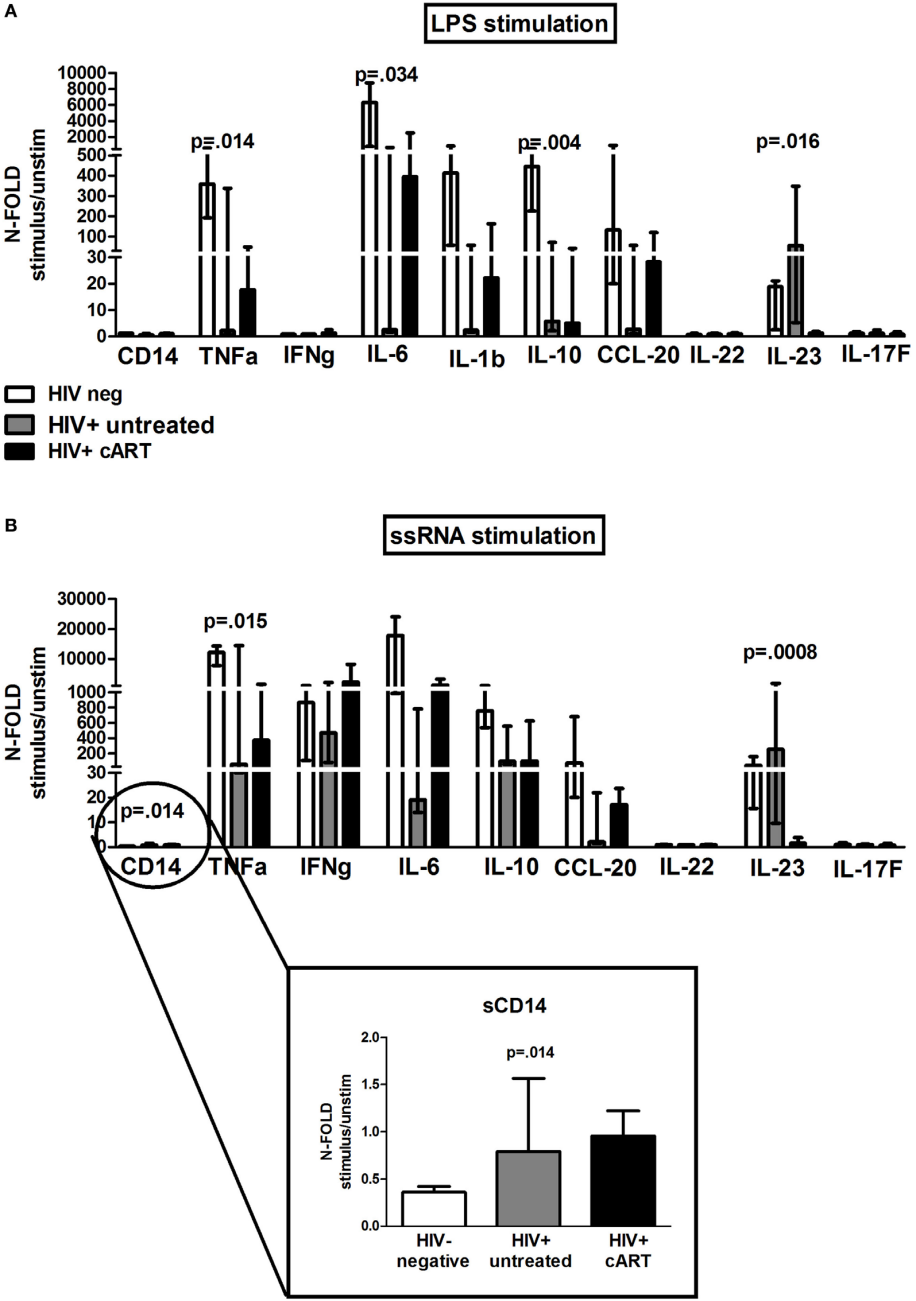
***In vitro* toll-like receptor challenge on PBMCs: cytokine/chemokine release**. 12 different analytes were measured from PBMCs supernatant of 5 HIV-negative healthy controls (white bars), 5 HIV+ untreated subjects (gray bars), and 11 HIV + combination antiretroviral therapy (cART) subjects (black bars) following lipopolysaccharide (LPS) and ssRNA, respectively. The figure depicts only cytokines/chemokines with a detectable production following LPS **(A)** and ssRNA **(B)** stimulation. Data shown as a fold change: (LPS-/ssRNA-stimulated cytokine value)/(unstimulated cytokine value). **(A)** LPS: PBMCs from untreated HIV+ produced significantly highest IL-23 (*p* = 0.016); PBMCs from cART-treated released higher TNF-α (*p* = 0.014), IL-6 (*p* = 0.034), IL-1b, and CCL-20 (*p* = 0.093) versus HIV+ untreated, lower than HIV-neg controls. **(B)** ssRNA: PBMCs from both HIV+ untreated and cART produced highest CD14 (*p* = 0.014), whereas HIV+ untreated patients released highest IL-23 (*p* = 0.0008). HIV + cART-treated patients tended to release higher TNF-α (*p* = 0.015), IFN-γ (*p* = 0.164), IL-6 (*p* = 0.067), and CCL-20 (*p* = 0.113) versus HIV+ untreated patients, lower, however, than uninfected controls.

Last, we assessed cytokine/chemokine levels in LPS/ssRNA-stimulated PBMC supernatants in a subset of 5 HIV-neg, 5 HIV+ untreated, and 11 HIV+ cART (5 INRs and 6 FRs), as proof of concept to be further investigated in larger cohorts (Figure 5).

Interestingly, in response to LPS, PBMCs from untreated HIV+ patients produced significantly higher IL-23 (*p* = 0.016) compared with cART-treated group, whereas PBMCs from cART-treated patients released higher TNF-α, IL-6, IL-1b, and CCL-20 as compared to untreated patients, however, lower than uninfected controls (TNF-α: *p* = 0.014; IL-6: *p* = 0.034; IL-1b: *p* = 0.330; CCL-20: *p* = 0.093; Figure 5A).

Viral stimulation accounted for greater release of cytokines/chemokines in all the study groups. In particular, upon ssRNA exposure PBMCs from both treated and untreated HIV-infected subjects produced highest CD14 (*p* = 0.014), whereas HIV+ untreated patients released highest IL-23 (*p* = 0.0008) (Figure 5B). HIV+ cART-treated patients tended to release higher TNF-α, IFN-γ, IL-6, and CCL-20 versus untreated patients, however, lower than uninfected controls (TNF-α: *p* = 0.015; IL-6: *p* = 0.067; IFN-γ: *p* = 0.164; CCL-20: *p* = 0.113; Figure 5B).

**Figure 6:**
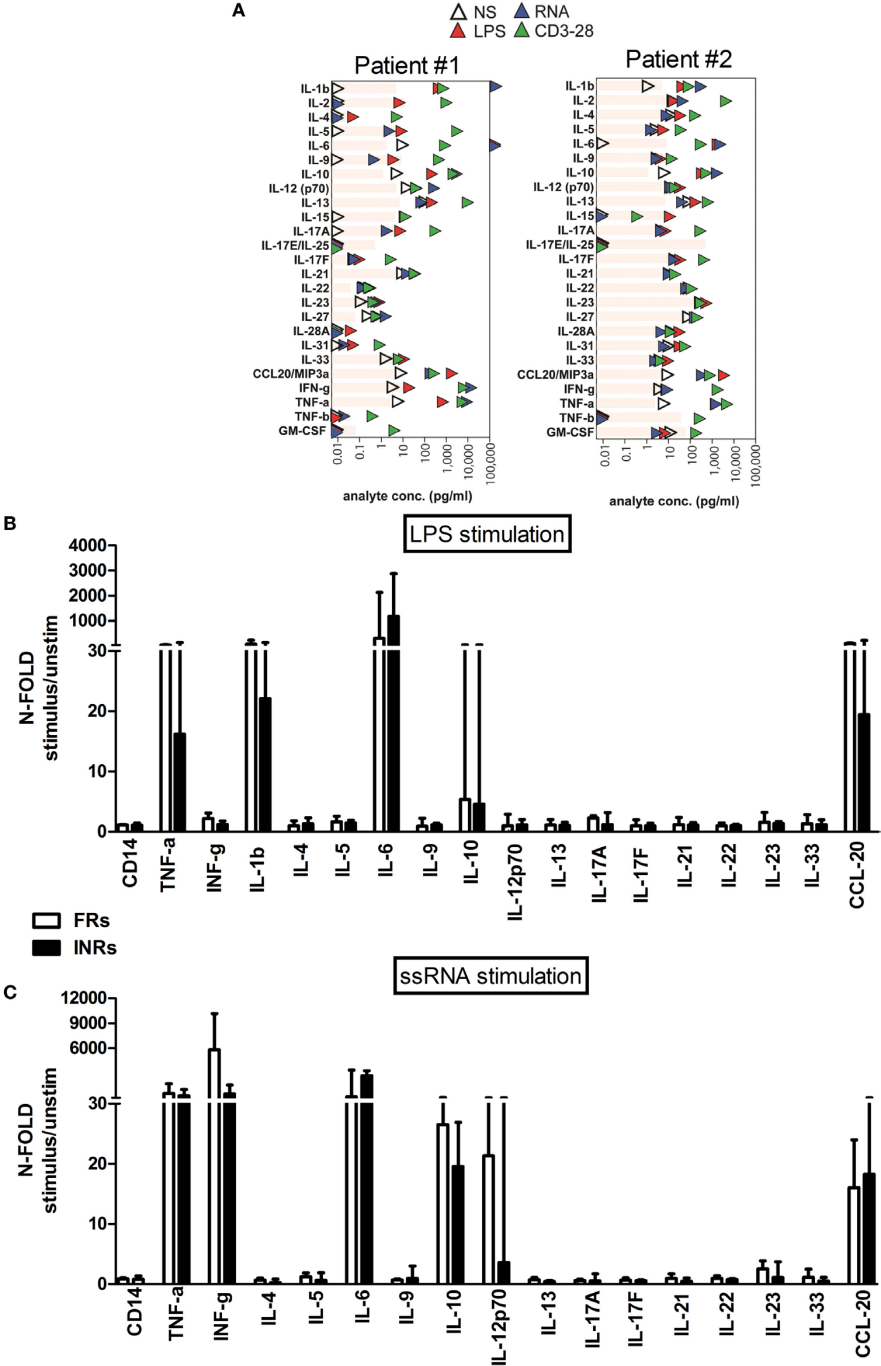
***In vitro* toll-like receptor challenge on PBMCs: cytokine/chemokine release in Full Responders (FRs) and Immunological Non-Responders (INRs)**. The 25 different analytes were measured from PBMCs supernatant of 5 INR and 6 FR patients following lipopolysaccharide (LPS) and ssRNA stimulation, respectively. The figure depicts only cytokines/chemokines with a detectable production following LPS **(A)** and ssRNA **(B)** stimulation. Data shown as a fold change: (LPS-/ssRNA-stimulated cytokine value)/(unstimulated cytokine value). **(A)** Each plot represents one individual, with the donor number noted at the top. In particular patient#1 is representative of FR group, whereas patient#2 is representative of INR group. The limits of detection of each analyte are shown in gray bars. **(B,C)** Data shown as a fold change: (LPS-/ssRNA-stimulated cytokine value)/(unstimulated cytokine value). **(B)** No significant differences in cytokine released between FRs and INRs after LPS exposure. **(C)** No major differences in cytokine release after ssRNA exposure.

However, when we stratified HIV+ cART patients according to immune recovery degree, there were no significant differences upon LPS and ssRNA exposure between FRs and INRs (Figure 6).

### Effect of TLR Stimulation on MDMs: HLA-DR/CD69 Cell-Surface Expression and Cytokine/Chemokine Release

Because our data on PBMC TLR challenge in HIV-infected patients failed to demonstrate major effects on the T-lymphocyte compartment, in the face of different patterns of cytokine/chemokine production according to presence or absence of therapy, we next sought to explore TLR challenge on an *in vitro* MDM system.

**Figure 7:**
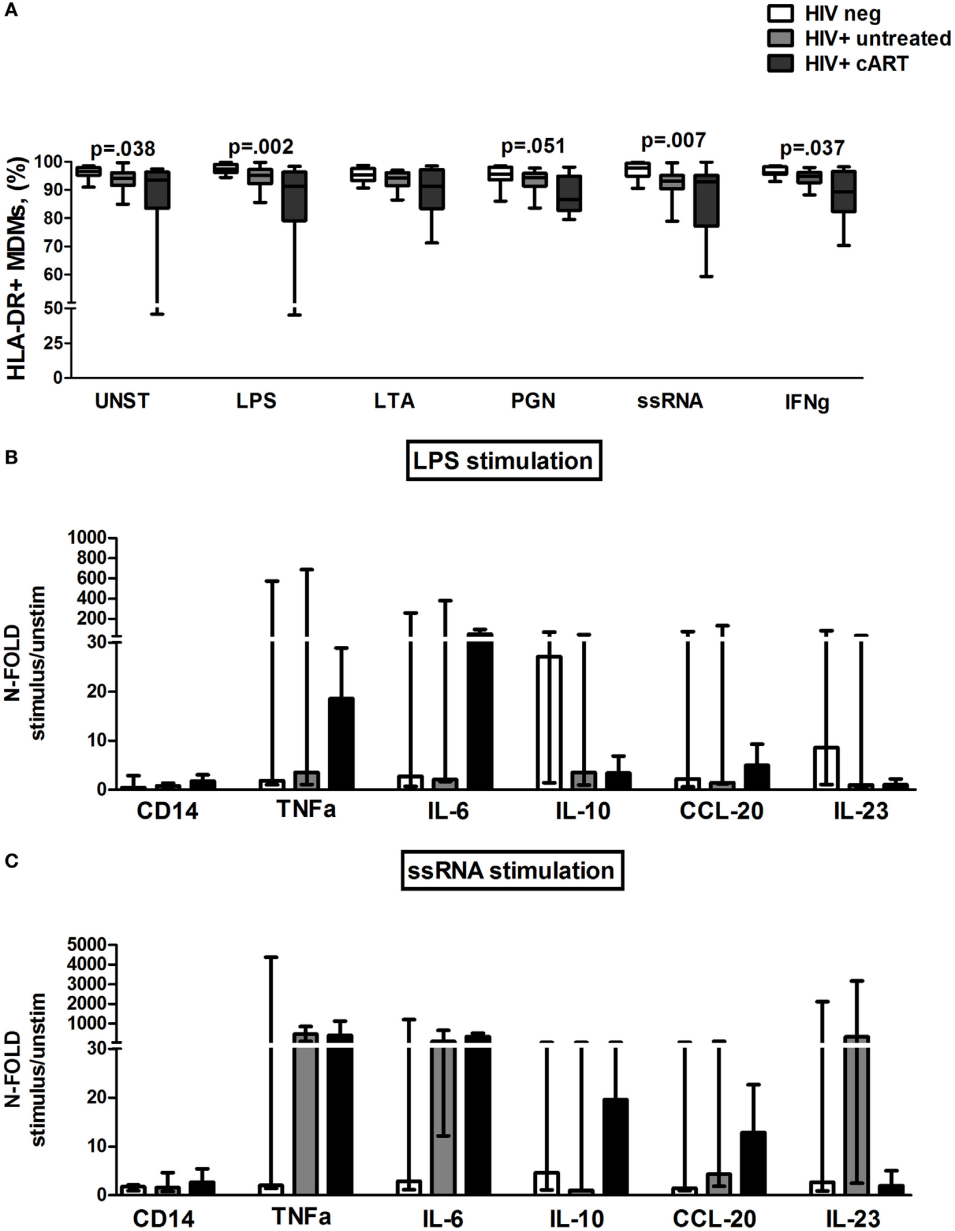
***In vitro* toll-like receptor (TLR) challenge on monocyte-derived macrophages (MDMs): HLA-DR expression and cytokine/chemokine release**. **(A)** Lower constitutively and TLR-driven HLA-DR expression on MDM in untreated and combination antiretroviral therapy (cART)-treated HIV+ versus HIV− controls. **(B,C)** Data presented as fold change (stimulated/unstimulated condition). Luminex quantification of 12 different analytes from MDM supernatant of 5 HIV-negative, 5 HIV+ untreated, and 5 HIV + cART subjects. **(B)** Feeble cytokine/chemokine release (<4-fold) from MDMs of untreated HIV+ after lipopolysaccharide (LPS) exposure. Higher TNF-α and IL-6 release in cART-treated patients following LPS challenge. **(C)** Higher cytokine/chemokine release, particularly TNF-α and IL-6 in both treated and untreated HIV-infected patients as compared to HIV− controls following ssRNA stimulation.

We first assessed the membrane expression of HLA-DR receptor. As expected, both treated and untreated HIV+ subjects consistently showed lower HLA-DR expression on MDM as compared to HIV-uninfected controls (Figure 7A).

We then studied MDM responsiveness to TLR challenge by the cell-surface expression of the activation marker CD69. Interestingly, in untreated HIV+ patients ssRNA stimulation was less efficient in increasing the proportion of CD69 + MDM as compared to HIV+ treated patients and HIV-negative controls (*p* = 0.015). No differences in both HLA-DR+ and CD69+ MDM were shown between INRs and FRs (data not shown).

To further investigate the effect of TLR stimulation on MDMs, cytokine and chemokine release was measured in supernatants following exposure to LPS and ssRNA (Figures 7B,C).

Interestingly, upon bacterial stimulation, MDMs of untreated HIV+ were characterized by lowest cytokine/chemokine release (<4-fold) (Figure 7B). Conversely, in cART-treated patients, LPS challenge raised TNF-α and IL-6 release despite not reaching statistical significance in comparison to the other groups (Figure 7B).

Viral stimulation accounted for greater cytokine/chemokine release, particularly TNF-α and IL-6 in both treated and untreated HIV+ subjects compared to HIV− controls (Figure 7C). No significant differences were found within cART patients following FRs and INRs categorization (data not shown).

Taken together, these findings point to *ex vivo* viral challenge as a more potent pro-inflammatory/activatory stimulus in both untreated and treated HIV when compared to bacterial stimulus. In particular, LPS failed to elicit strong immune responses in untreated individuals, suggesting tolerance to already elevated endotoxemia in these patients.

## Discussion

Our report sought to help elucidate the role of signaling *via* TLR pathways in sustaining immune activation in cART-treated HIV-infected patients as compared to both HIV+ untreated patients and uninfected controls.

Despite complete or near-complete suppression of HIV replication with cART, chronic inflammation and immune activation persist indefinitely (54, 55) and have been implicated in the pathogenesis of both impaired immunological recovery (1–4) and clinical outcome (8, 9, 56, 57).

Therefore, dissecting the mechanisms that sustain such persistent chronic immune activation and its relationship with poor immune reconstitution will provide important avenues to improve disease management in HIV-infected cART-treated individuals.

Prior *in vivo* studies have shown an association between microbial translocation, immune activation, and inadequate CD4 recovery on cART (32, 58). Along the same line, *in vitro* research has shown a direct link between microbial stimulation and immune activation in both HIV-uninfected (12, 37–39, 53) and HIV-infected untreated individuals through TLR signaling (18, 31, 33, 50, 59–61).

We, first, assessed the response of PBMCs to bacterial and viral TLR stimulation in all study groups. Upon bacterial (LPS) stimulation, PBMCs from both treated and untreated patients displayed an overall feeble cytokine release, possibly reflecting tolerance to the highest levels of circulating LPS or recent *in vivo* activation. PBMCs from cART patients featured greater production of cytokines/chemokines directly involved in inflammation, such as TNF-α, IL-6, IL-1b, and CCL-20 (yet lower than healthy HIV-uninfected controls). Conversely, PBMCs from antiretroviral-naïve patients released highest IL-23, a finding consistent with previous research and that has been correlated with systemic immune activation and gut damage (36, 62).

Similar findings were manifested in an experimental setting of TLR-stimulated MDMs from cART-treated patients, which displayed lowest HLA-DR expression and highest LPS-driven release of pro-inflammatory TNF-α and IL-6, altogether indicating severely impaired monocyte/macrophage function with a broader pro-inflammatory potential, reminiscent of what is observed in severe pro-inflammatory clinical settings (63).

Interestingly, in our patients’ cohort, none of the bacterial agonists that we tested and that mimic both Gram+ and Gram− bacteria (i.e., LPS, LTA, PGN) resulted in a significant expansion of activated, proliferating, apoptotic T-lymphocytes, confirming the modest effect of TLR stimulation alone in the direct activation of T-lymphocytes (37, 38).

We, next, evaluated the PBMC/MDM response to viral stimulation, which proved a stronger pro-inflammatory challenge in HIV-infected individuals as a whole. Despite full HIV-viremia suppression, TLR7/8 stimulation, which has been shown to recognize HIV-1 ssRNA, resulted in high transcription of genes involved in the TLR pathway and pro-inflammatory cytokine/chemokine release from PBMCs and MDMs of cART-treated patients, at levels not substantially lower than HIV+ untreated patients. Most interestingly, direct ssRNA stimulation resulted in a significant expansion of activated CD38+ CD8 T-cells uniquely in cART patients.

Two major observations stem from the findings of this first part of the study: (i) viral stimulation appears a stronger stimulus than LPS, clearly pointing to HIV itself, or its components, as a major source of innate immune activation even in the presence of virally suppressive cART, as also suggested by the demonstration in our patients of low-level residual viremia, at levels below the threshold of commercially available assays. (ii) Bacterial stimulation seems to exert a superior effect on monocyte/macrophage activation from cART-treated *versus* untreated patients, with no major effect on T-lymphocytes, pointing to translocating bacteria as selective stimulus to innate immunity during virally suppressive cART. Because clinical and translational research strongly suggests that chronic activation of innate immunity drives morbidity/mortality in treated HIV (64–68), our data provide *in vitro* evidence (tested in culture from both whole PBMCs and MDMs) of how monocyte/macrophages from cART patients might be preferentially activated by circulating bacterial products. Given the persistence of gut damage and microbial translocation during cART (9, 25, 26, 29, 69), we hereby provide an indication of how the systemic exposure to bacterial TLR agonists translocated from a damaged gut may contribute to excessive morbidity/mortality in these patients.

In the second part of our research, we restricted the analysis to cART-treated patients with different CD4 recovery, with the ultimate aim to dissect possible role of TLR signaling in the inadequate CD4 reconstitution on cART. Despite our approach of gene expression analysis does not capture a statistical significance also due to the small number of patients investigated, PBMCs from INRs seem to display greater constitutive mRNA expression of genes involved in the TLR-mediated pathway, yet upregulated only type I IFN genes following stimulation with TLR agonists. In contrast, TLR engagement in FRs seemed to result in the broad induction of genes involved in innate and adaptive immune responses. Taken together, these findings may suggest less efficient TLR-mediated signaling following exposure to TLR ligands in individuals with poor immune recovery after cART, which may be explained by tolerance (70, 71) or achievement of maximal transcription levels. Furthermore, by suggesting higher transcription of type I IFN genes in INRs in response to viral challenge, our findings support further investigation of IFN/interferon-stimulated genes pathways, both of which have been proven to accelerate HIV/SIV pathogenesis, according to the response to cART (72–74).

In addition, while our failure to find any differences between INRs and FRs in cytokine/chemokine release by PBMCs and T-cell activation might be due to the limited number of patients analyzed, it may also reflect the mixed contribution of TLR-dependent and -independent pathways in supporting immune activation and inflammation.

Several caveats in the experimental design of the present study must be acknowledged including prolonged cell culture, which might have selected the strongest and most functional MDMs, and the lack of cocultures of single APCs (macrophages, dendritic, or B cells) and CD3+ T-cell subpopulation, which could have more precisely highlighted the contribution to TLR signaling pathways of each cell subset. Despite these limitations, we show an impairment in TLR pathway of HIV-infected subjects that seems to persist upon cART long-term treatment with possible differences according to the degree of immune recovery. Bacterial TLR agonists have little effect on MDM-mediated signaling, implying a role of other cell populations in inducing T-cell activation in this setting. Viral components nonetheless appear to affect MDM activation in all subjects receiving cART.

Taken together, our findings indicate systemic exposure to microbial TLR agonists as driver of immune activation in treated HIV and suggest that the contribution of persistent (low-level) viral exposure might overweight microbial exposure. In this view, our research may partially facilitate an explanation of why rifaximin or sevelamer, by antagonizing microbial bioproducts in the systemic circulation, may not lower T-cell activation in HIV-infected humans (75, 76), despite the intriguing results obtained in the animal model (77). The research may also suggest the need for a more comprehensive elucidation of the role of the TLR system in promoting HIV-driven immune hyperactivation. Challenges include a broad analysis of the differential role of TLR-mediated signaling and the exact set of regulators of TLR pathway (e.g., miRNAs), as well as genes/molecules activated upon TLR stimulation within different cell subpopulations and their downstream effector functions. Unraveling the role of the TLR pathway(s) in orchestrating immune activation in the context of treated infection is mandatory for its exploitation for therapeutic purposes. Finally, the observation of cytokine release from MDMs of HIV-infected subjects pose monocytes/macrophages as an important source of pro-inflammatory mediators, which have been associated with cardiovascular disease, HIV-associated neurocognitive development, and innate immune aging (78).

Thus, therapeutically targeting sources and pathways of monocyte activation could be a useful strategy to limit immune activation associated with the premature development of age-related diseases for HIV-infected persons treated with cART.

## Ethics Statement

This study was carried out in accordance with the recommendations of Ethical Committee of ASST “Santi Paolo e Carlo”, Milan, Italy with written informed consent from all subjects. All subjects gave written informed consent in accordance with the Declaration of Helsinki. The protocol was approved by the Comitato Etico, ASST “Santi Paolo e Carlo”, Milan, Italy.

## Author Contributions

EM designed the study, designed and performed experiments, analyzed and interpreted the data, designed the figures, and wrote the manuscript. MB, IS, FC, AC, and JS-C performed the experiments and analyzed the data. CT analyzed and interpreted the data and wrote the manuscript. MC and AdM helped with interpreting the results and edited the manuscript. GM conceived and designed the study, interpreted the data, and wrote the manuscript.

## Conflict of Interest Statement

EM reports other from Gilead Italia, outside the submitted work. AC reports other from MilliporeSigma, outside the submitted work. GM reports other from janssen-cilag, grants from Gilead, personal fees from Gilead, AbbVie, and Janssen-Cilag, outside the submitted work. CT reports non-financial support from Janssen-Cilag, personal fees from BMS, personal fees from Merck, Gilead, AbbVie, and Janssen-Cilag, outside the submitted work. The other authors declare that the research was conducted in the absence of any commercial or financial relationships that could be construed as a potential conflict of interest.
